# Improved Diabetes Control and Pancreatic Function in a Type 2 Diabetic after Omeprazole Administration

**DOI:** 10.1155/2012/468609

**Published:** 2012-03-14

**Authors:** I. N. Mefford, J. T. Mefford, C. A. Burris

**Affiliations:** Fort Bend Premier Care, 1505 Liberty Street, Richmond, TX 77469, USA

## Abstract

A 43-year-old man with type 2 diabetes, opposed to insulin use and poorly responsive to oral agents added sequentially over 6 years, was placed on 40 mg omeprazole twice daily. A linear decline in daily fasting blood glucose was observed over the first two-month treatment, and his hemoglobin A1c was reduced from 11.9% to 8.2%, then sustained at 8.1% after four months. Glucose, insulin, and C-peptide response to a 2-hour glucose tolerance test were consistently improved across this time period, and calculated beta-cell mass increased by 67%. We believe these responses are consistent with activation or neogenesis of pancreatic beta cells, possibly through a gastrin-mediated mechanism.

## 1. Introduction

Type 2 diabetes is a progressive disease characterized by both insulin resistance and increasing dysfunction of pancreatic beta cells, either through inactivation or apoptosis [1, 2]. Common treatments of type 2 diabetes may modify insulin sensitivity, increase insulin secretion, or in some cases either reduce beta-cell dysfunction or slow their degradation [3]. However, none of the current available agents are known to increase beta-cell population in humans. Meier et al. have demonstrated increased beta-cell activity in the human pancreas surrounding gastrinomas [4], and further studies have shown gastrin administration to induce pancreatic beta-cell neogenesis in animal models of diabetes [5, 6]. We previously observed in a retrospective analysis of our patient database a significant improvement in type 2 diabetes control in patients concurrently taking proton pump inhibitors (PPIs) [7]. These data are supported by similar observations that have since been made in three other independent data sets [8–10]. PPI administration has been shown to reverse diabetes in a murine model when combined with a dipeptidyl peptidase (DPP-4) inhibitor [11] and to markedly improve glycemic control in *Psammomys obesus*, a model for type 2 diabetes [12]. PPIs are known to induce hypergastrinemia [13], and such an effect has been hypothesized to mediate these observations.

We now report a case study of a 43-year-old man with uncontrolled type 2 diabetes, treated consecutively and concurrently for several years with multiple oral agents, who had declined to be advanced to insulin use. In lieu of starting insulin administration and based on earlier observations in our database, the patient was placed on omeprazole 40 mg twice daily; measures of pancreatic insulin secretion and diabetes control were obtained across a four-month period.

## 2. Case Report

### 2.1. Patient Background

A 43-year-old man with type 2 diabetes, compliant with medications but poorly controlled, had been followed in our clinic for 6 years. He was diagnosed with diabetes in 2003, and his hemoglobin A1c (HgbA1c) had ranged between 10.4 to 12.3% throughout that time (11.4% ± 0.4) in spite of oral medications and weight loss. He had been taking and continued to take oral diabetes medications, Metformin 1000 mg twice daily, Glipizide 10 mg daily, Actos (pioglitazone) 45 mg daily, and Januvia (sitagliptan) 100 mg daily. In addition, he carried diagnoses of dyslipidemia and hypertension, taking Lipitor 10 mg and Lisinopril 20 mg daily.

### 2.2. Treatment

During the treatment period, the patient did not markedly vary in weight and had no remarkable change in diet or exercise. No routine medications were either started or discontinued, and compliance with medication regimen was determined by patient interview. Oral treatment of omeprazole, 40 mg, twice daily, was added to his daily regimen to obtain sustained reduction in gastric acidity and sustained elevation in serum gastrin. He continued to record fasting AM blood glucose throughout the test period and continued on all other medications.

### 2.3. Laboratory Measurements

HgbA1c, fasting glucose, gastrin, insulin, and C-peptide levels were obtained in a fasting state prior to starting the omeprazole treatment and at 1 and 2 hours following a standard glucose tolerance test (GTT). All laboratory measurements were performed by a commercial laboratory (Quest Diagnostics), and samples were collected by a professional phlebotomist employed by Quest Diagnostics in their standardized fashion. Daily fasting glucose measurements were obtained with a commercial glucometer by the patient and recorded. After 2 and 4 months the patient returned to the clinic, in a fasting state, having taken no medications on the morning of testing for repeat measurements of HgbA1c, gastrin, and a 2-hour GTT measuring glucose, insulin, and C-peptide.

### 2.4. Calculations

Statistical analysis was performed with commercial statistical software, “Analyse-It for Microsoft Excel v2.20”. Calculation of pancreatic beta-cell mass by HOMA was performed by the method of Matthews et al. [14], and an alternative calculation of beta-cell area was performed using 2-hour glucose after GTT by the method described by Meier et al. [15].

## 3. Results

**Figure 1:**
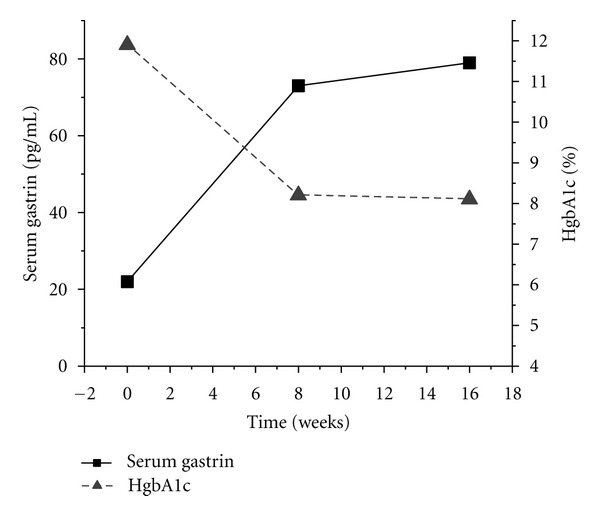
Effect of twice daily 40 mg omeprazole treatment on serum gastrin and hemoglobin A1c in a type 2 diabetic.

A nearly linear decline in daily fasting blood glucose was observed throughout the first 2 months of the trial period (*y* = −0.672*x* + 27064, *r* = −0.35, *P* = 0.0102, data not shown). Initial fasting blood glucose, 240 mg/dL at the start of treatment, declined to 138 mg/dL at the end of 8 weeks. HgbA1c, 11.9% at the start of omeprazole administration, decreased to 8.2% at the end of 8 weeks and 8.1% at the end of 16 weeks. Serum gastrin, 22 pg/mL prior to omeprazole administration, increased to 73 pg/mL at 8 weeks and 79 pg/mL at the end of 16 weeks of omeprazole administration (see Figure 1).

**Figure 2:**
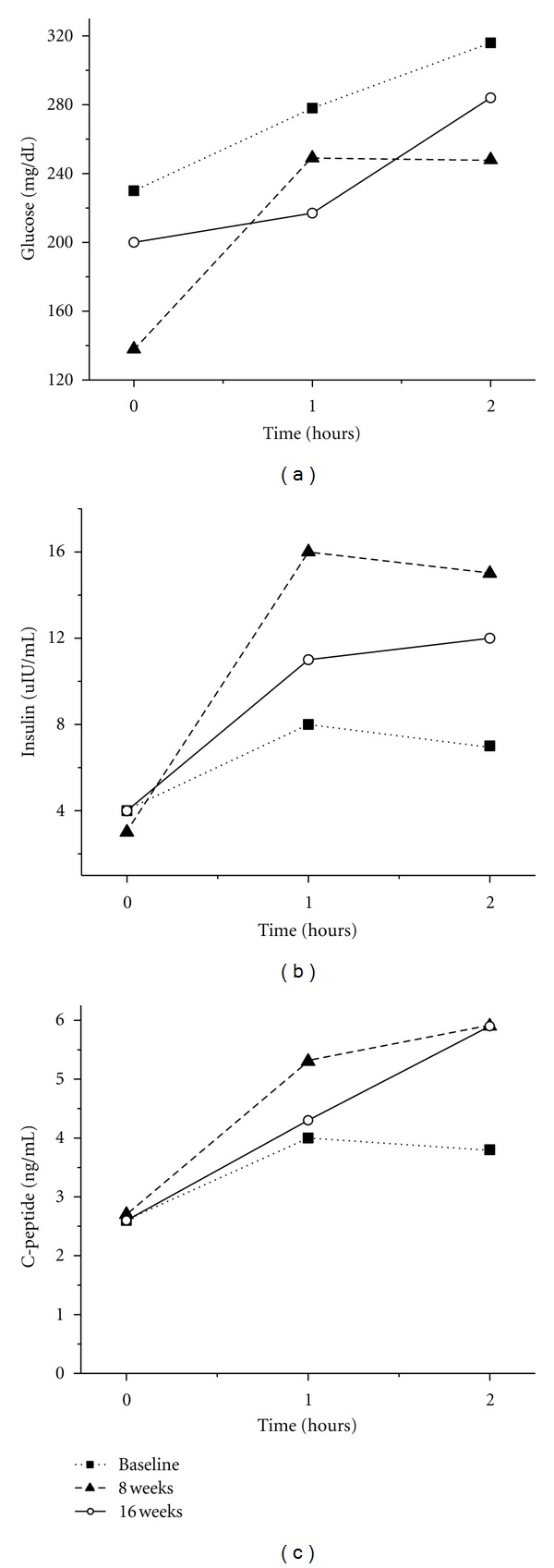
2 hr glucose tolerance test effects on blood glucose (a), insulin (b), and C-peptide (c) after twice daily 40 mg omeprazole treatment in a type 2 diabetic.

Figure 2 shows the results of the 2-hour GTT on glucose, insulin, and C-peptide. Fasting, maximal, and 2-hour glucose values were markedly decreased after 8 weeks of omeprazole (Figure 2(a)). Figures 2(b) and 2(c) reveal a marked increase in the insulin and C-peptide secretion after glucose stimulation, nearly doubled in each case, consistent with activation or neogenesis of pancreatic beta cells.

Using the HOMA method for calculation of beta-cell mass [14], a 67% increase in beta-cell mass was found. This is comparable to a 69% increase in beta-cell area, 0.21 to 0.36, using the method of Meier et al. [15].

## 4. Discussion

We have observed and previously reported improved glucose control in type 2 diabetes in patients taking PPIs [7, 8]. Two additional groups have confirmed these observations in independent patient populations [9, 10]. Preclinical studies have shown that PPI administration can induce beta-cell neogenesis and improve diabetes control in an animal model of type 2 diabetes [11].

 In our initial report, much of the difference between patients on PPIs and those not taking PPIs could be accounted for in the differences found in patients also taking a secretagogue such as a sulfonylurea. In these patients, the reduction in HgbA1c was much greater, 1.5% (*P* < 0.001), compared to the overall group which showed a reduction of 0.6% (*P* = 0.002) [7]. In our experience since then, PPI administration appears to have a greater effect on those patients concomitantly taking a secretagogue, either a sulfonylurea or a meglitinide. It is unclear if duration of diabetes influences these effects, and in our clinical observations, a smaller beta-cell population as seen in the present patient may allow for a relatively larger effect. For example, an increase from 0.21 to 0.36 percent beta-cell area is a much greater relative effect than might be recognized in a diabetic with a basal beta-cell area of one percent having an equal 0.15 percent increase. It was because of these observations that the present treatment regimen was undertaken.

PPI administration can have a variety of metabolic effects, including delayed gastric emptying [16, 17], which might account for a portion of the improved glycemic control, including the lower stimulated maximal glucose in the 2-hour GTT. This should not, however, account for increased insulin and C-peptide secretion. Alternate effects of omeprazole on somatostatin or other antral peptides might alter glucagon or insulin secretion [18, 19] and may explain the observed effects on insulin or C-peptide secretion. In fact, a combination of effects might result in our observations. We are unable to speculate on possible effects of PPIs on incretins as we are unaware of any interaction at this time, although such an interaction might have relevance. Regardless of the actual mechanism, the present case study is consistent with improved pancreatic beta-cell function.

A 43-year-old man with poorly controlled diabetes who had been stable on all medications for approximately 1 year prior to the omeprazole administration showed consistent elevation of gastrin throughout the trial period and a marked (3.8%) reduction in HgbA1c through the same period. Markers of pancreatic function, insulin, and C-peptide secretion after glucose administration (2 hr GTT) were consistent with a more robust pancreatic response, supported by a lower stimulated maximal glucose after 2 hr GTT. These data are consistent with either improved pancreatic beta-cell function or neogenesis of beta cells, possibly mediated by the sustained elevation in gastrin. It is possible that the lower maximal glucose increase after the 2 hr GTT may be explained by improved insulin sensitivity, delayed gastric emptying, or an effect of omeprazole on a mediator of glucose or insulin metabolism; however, these mechanisms would not explain the improved insulin and C-peptide secretions. Further studies are needed to elucidate the mechanism of these effects.
